# Sonazoid-enhanced ultrasound for preoperative localization and characterization of sentinel lymph nodes in patients with cutaneous melanoma

**DOI:** 10.1007/s00330-026-12640-2

**Published:** 2026-05-25

**Authors:** Binbin Jiang, Liqi Sun, Lu Si, Jiayong Liu, Chuanliang Cui, Kun Yan, Shanshan Yin

**Affiliations:** 1 Department of Ultrasonography, Key Laboratory of Carcinogenesis and Translational Research (Ministry of Education/Beijing), Peking University Cancer Hospital & Institute, Beijing, China; 2 Department of Ultrasound, The Second Hospital Affiliated with the Southern University of Science and Technology (Shenzhen Third People’s Hospital), Shenzhen, China; 3 Department of Melanoma and Sarcoma, Key Laboratory of Carcinogenesis and Translational Research (Ministry of Education/Beijing), Peking University Cancer Hospital and Institute, Beijing, China; 4 Department of Bone and Soft Tissue Tumor, Key Laboratory of Carcinogenesis and Translational Research (Ministry of Education/Beijing), Peking University Cancer Hospital & Institute, Beijing, China; 5 Peking University Cancer Hospital (Inner Mongolia Campus) & Affiliated Cancer Hospital of Inner Mongolia Medical University, Huhhot, China

**Keywords:** Ultrasonography, Sentinel lymph node biopsy, Melanoma, Lymphatic metastasis, Sonazoid

## Abstract

**Objective:**

Accurate sentinel lymph node (SLN) identification is essential for the staging of cutaneous melanoma. Contrast-enhanced ultrasound using Sonazoid (Sonazoid-CEUS), which is based on a perfluorobutane microbubble contrast agent with reticuloendothelial uptake properties, was evaluated for SLN detection in patients with stage Ib–II melanoma.

**Materials and methods:**

In this prospective single-arm cohort study, 71 eligible subjects underwent perilesional intradermal Sonazoid-CEUS, followed by lymphoscintigraphy and SLN biopsy. The numbers of SLNs detected via CEUS and lymphoscintigraphy were compared by using non-parametric paired analysis (Wilcoxon signed-rank test). The diagnostic accuracy of CEUS for metastatic involvement was evaluated against histopathological results.

**Results:**

Sonazoid-CEUS achieved a 97.3% success rate in identifying SLNs, detecting 148 SLNs, with an average of 2.1 ± 1.1 SLNs per patient. Most patients (93.0%) had single-basin lymphatic drainage. For metastasis detection, CEUS demonstrated 82.1% sensitivity, 88.4% specificity, and 85.9% accuracy. Ulceration, high mitotic count, and HMB45 positivity in primary lesions were significantly associated with SLN metastasis. No contrast-related adverse events were observed.

**Conclusion:**

Sonazoid-CEUS is a safe and accurate non-radioactive technique for SLN identification and preoperative diagnosis in patients with cutaneous melanoma; moreover, it exhibits potential value as a complementary imaging modality within established SLNB workflows.

**Key Points:**

***Question*** *Can a non-radioactive contrast-enhanced ultrasound technique using Sonazoid improve preoperative localization and characterization of sentinel lymph nodes in patients with cutaneous melanoma?*

***Findings*** *In this prospective study, Sonazoid-CEUS identified sentinel lymph nodes in 97.3% of patients and showed high diagnostic accuracy without contrast-related adverse events.*

***Clinical relevance*** *Sonazoid-CEUS provides a non-radioactive adjunct for preoperative sentinel lymph node mapping and characterization in cutaneous melanoma, thereby potentially improving surgical planning while offering complementary functional information alongside conventional lymphoscintigraphy.*

**Graphical Abstract:**

**Figure:**
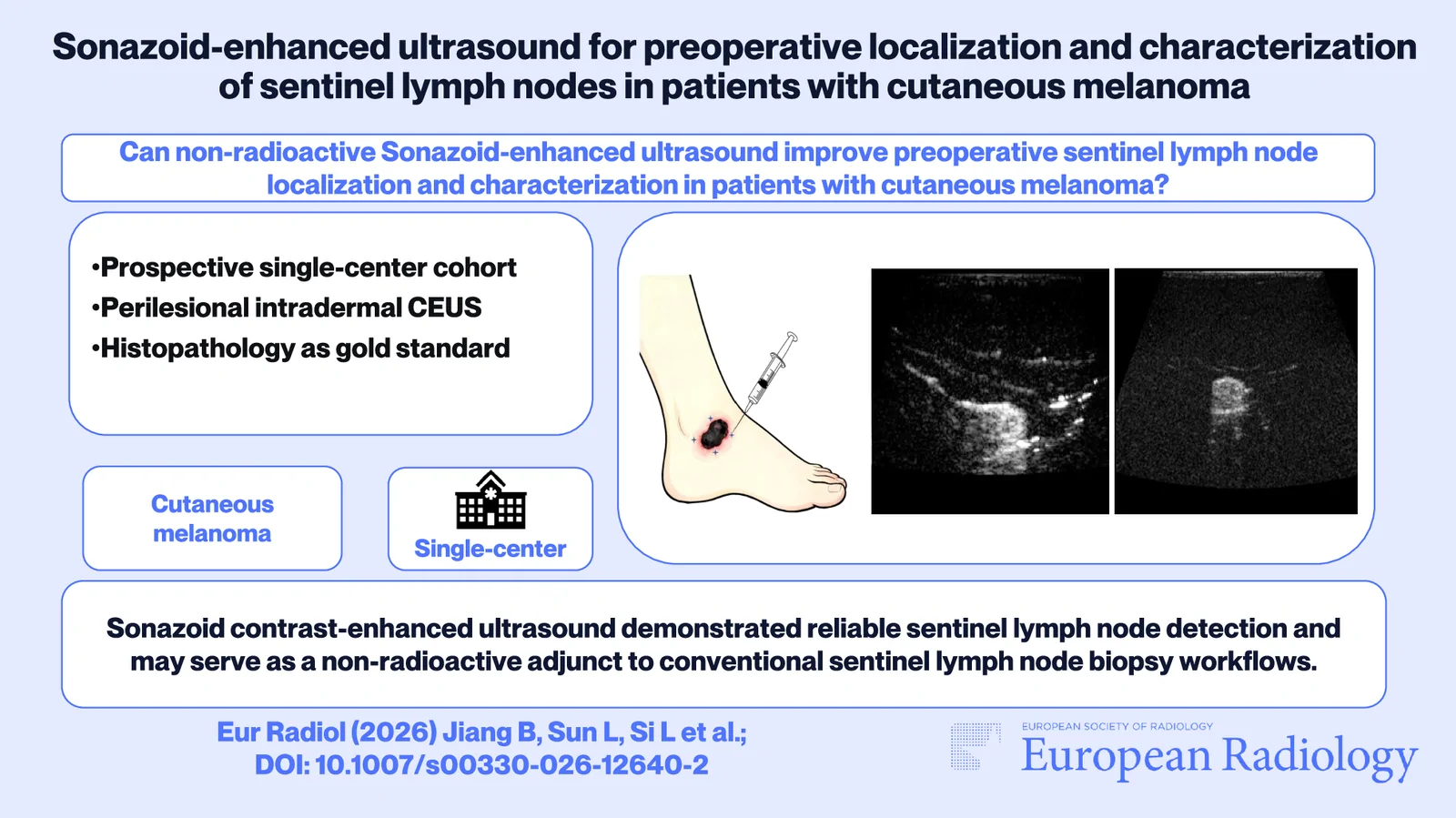

## Introduction

Cutaneous malignant melanoma is one of the most common skin malignancies, with increasing incidence rates being reported worldwide [1–3]. Regional lymph nodes are the most common site of melanoma metastases and are often the first site involved in metastatic disease [4]. The sentinel lymph node (SLN), defined as the first lymph node station, serves as a critical determinant for tumor staging, prognosis evaluation, and subsequent treatment planning [4, 5]. Therefore, the accurate identification of SLNs is essential for melanoma staging and treatment [6]. Sentinel lymph node biopsy (SLNB) has become the standard staging procedure for clinically node-negative patients and provides vital prognostic information [7–9].

Currently, SLN localization primarily relies on lymphoscintigraphy, which uses radioactive isotopes to identify the first lymph node in the tumor area, in combination with gamma probes and/or dye for localization [10]. Lymphoscintigraphy has been observed to exhibit high sensitivity in SLN detection, particularly in melanoma staging [11]. In recent years, the combination of single-photon emission computed tomography/computed tomography (SPECT/CT) imaging with lymphoscintigraphy has significantly improved SLN detection accuracy, particularly in complex anatomical areas such as the head and neck [12]. However, traditional radioactive isotope and blue dye (such as methylene blue and Patent Blue V) tracers have certain limitations, such as the potential to cause allergic and skin reactions, as well as radiation risks and the need for expensive detection equipment [13–16].

Compared to traditional methods, contrast-enhanced ultrasound (CEUS) technology, which demonstrates advantages such as high resolution, simple operation, lack of radiation, low cost, and accurate positioning, overcomes the deficiencies of traditional tracers to a certain extent [17]. Among ultrasound contrast agents, SonoVue (Bracco) has been relatively well established, with clinical studies confirming its favorable accuracy and safety in SLN identification in patients with melanoma [18, 19]. However, as it is a pure blood-pool imaging agent, its short duration of contrast enhancement hinders prolonged observation [20]. Recently, Sonazoid (GE Healthcare) has been used as a second-generation, microbubble-based ultrasound contrast agent. It is primarily used in Japan and Korea for diagnosing hepatocellular carcinoma; moreover, it is useful in this setting because of its unique uptake in Kupffer cells. This agent is composed of perfluorobutane microbubbles stabilized by hydrogenated egg phosphatidylserine sodium [21]. Sonazoid not only demonstrates high tolerance to ultrasound mechanical indices but also is selectively phagocytosed by the reticuloendothelial system within lymph nodes. This property enables prolonged retention in the lymphatic tissue and increases the signal-to-noise ratio, thereby offering superior imaging quality and diagnostic reliability [22]. As a result, Sonazoid provides a significant advantage in SLN localization by enhancing SLN visualization [23–25]. These emerging imaging technologies may address the limitations of traditional methods, optimize preoperative staging, and reduce the risk of invasive surgery for patients.

Therefore, this study aimed to systematically evaluate the feasibility and clinical safety of the novel ultrasound contrast agent, known as Sonazoid, in SLN identification for cutaneous malignant melanoma. By comparing the performance of Sonazoid with that of existing techniques, we seek to delineate its clinical advantages and utility, thereby providing a more reliable imaging tool for lymphatic staging in melanoma.

## Materials and methods

### Study design and population

This prospective, single-arm cohort study was approved by the institutional ethics committee, and all patients provided informed consent prior to participation. Patients were eligible if they met the following inclusion criteria: (1) pathologically confirmed stage IB or II primary cutaneous or acral malignant melanoma and (2) confirmation of the absence of distant metastasis and clinical regional lymph node metastasis on relevant staging examinations prior to CEUS. The exclusion criteria were as follows: (1) absence of lymphoscintigraphy; (2) absence of enhanced lymphatic channels detectable on CEUS; (3) allergy to eggs; and (4) pregnancy or lactation.

### CE-SLN

In this study, CEUS was performed using ultrasound diagnostic systems (GE Logiq E9 and Siemens S3000 series) equipped with CEUS capabilities combined with high-frequency transducers for superficial organ CEUS examinations. The microbubble contrast agent, known as Sonazoid, was dissolved in 2 mL of saline (0.9% NaCl) before use. A total of 0.2 mL of the solution was administered via perilesional intradermal injection at the 12, 3, 6, and 9 o’clock positions, which were placed within approximately 5 mm of the lesion margin or surgical scar. The injections were deliberately confined to the dermal layer to ensure entry into the superficial dermal lymphatic plexus [26, 27], which is consistent with established technical principles for sentinel lymph node mapping in melanoma and for minimizing drainage toward non-first-echelon nodes. Gentle manual massage of the injection site was performed for 20–30 s to facilitate lymphatic uptake.

After the injection and massage, another experienced sonographer operated the probe, switched the instrument to CEUS mode, and used a real-time dual-image display to identify the SLN. The perilesional region was carefully scanned to identify enhancing lymphatic channels (LCs). Once an enhancing LC was visualized, the probe was advanced along its course to dynamically track lymphatic flow toward the regional nodal basin. The first lymph node demonstrating contrast enhancement along each traced lymphatic channel was defined as the sentinel lymph node (SLN). If multiple lymphatic vessels were identified, each vessel was independently tracked to determine separate drainage pathways. This real-time visualization and tracking of enhancing lymphatic vessels enabled the functional identification of first-echelon nodes rather than secondary nodal stations. The number, size, anatomical location, and enhancement pattern of the SLNs were recorded, and the course of the enhanced lymphatic vessels and the location of the SLNs were marked on the body surface.

CEUS images were interpreted via consensus by two radiologists with over 5 years of experience in lymphatic contrast-enhanced ultrasound who were blinded to clinical and pathological data, with disagreements being resolved via joint review. The enhancement pattern of the identified SLNs was classified into three types based on the homogeneity of microbubble perfusion within the node: Type I (homogeneous enhancement), Type II (inhomogeneous enhancement with focal perfusion defects), and Type III (no enhancement or rim enhancement only). For the purpose of qualitative diagnosis, Type I was considered to be indicative of a benign node, whereas Types II and III were considered to be suspicious for metastasis.

If no lymphatic vessel enhancement was observed, the massage was repeated. If enhancement still did not occur, the contrast agent was reinjected. The absence of lymphatic enhancement after two consecutive injections was considered an examination failure. Patients were monitored for clinical symptoms and vital signs at 15 min, 30 min, and 1 h after administration of the ultrasound, and a follow-up evaluation was conducted 24 h later to record any adverse reactions.

### Lymphoscintigraphy and surgical procedure

All patients received an intradermal administration of 99mTc-rituximab (11.1–18.5 MBq in 0.4 mL) 2 to 6 h prior to surgery. Lymphoscintigraphy was performed 0.5 to 1 h after injection using a SPECT/CT instrument (Siemens Symbia). Patients were placed in the supine position, and anterior planar images were acquired to identify SLNs and determine their number.

During SLNB, SLNs were localized with a handheld gamma probe (Neoprobe 2000) based on preoperative SPECT. The resected SLNs underwent histological evaluation via haematoxylin and eosin (H&E) staining (discontinuous sectioning, 10 sections per case), supplemented with immunohistochemical staining for S-100, HMB45, and Melan-A.

### Statistical analysis

In this study, the success rate of CEUS for SLN identification was defined as the proportion of patients in whom at least one first-echelon enhancing lymph node was identified following perilesional intradermal contrast administration, with real-time visualization of the afferent lymphatic channels from the injection site being demonstrated. Given the count nature of the data, the normality of the differences in SLN counts was assessed by using the Shapiro–Wilk test. As the distribution deviated from normality, the primary comparison was performed by using the non-parametric Wilcoxon signed-rank test, with the paired *t*-test being applied as a supplementary analysis to assess the robustness of the results. The diagnostic performance of CEUS for metastatic involvement was assessed by using histopathological results as the gold standard. Various metrics were calculated, including sensitivity, specificity and accuracy. A *p*-value < 0.05 was considered to indicate statistical significance. All the statistical analyses were performed using SPSS version 22.0 (IBM Corp.).

## Results

### Patient and tumor characteristics

**Fig. 1:**
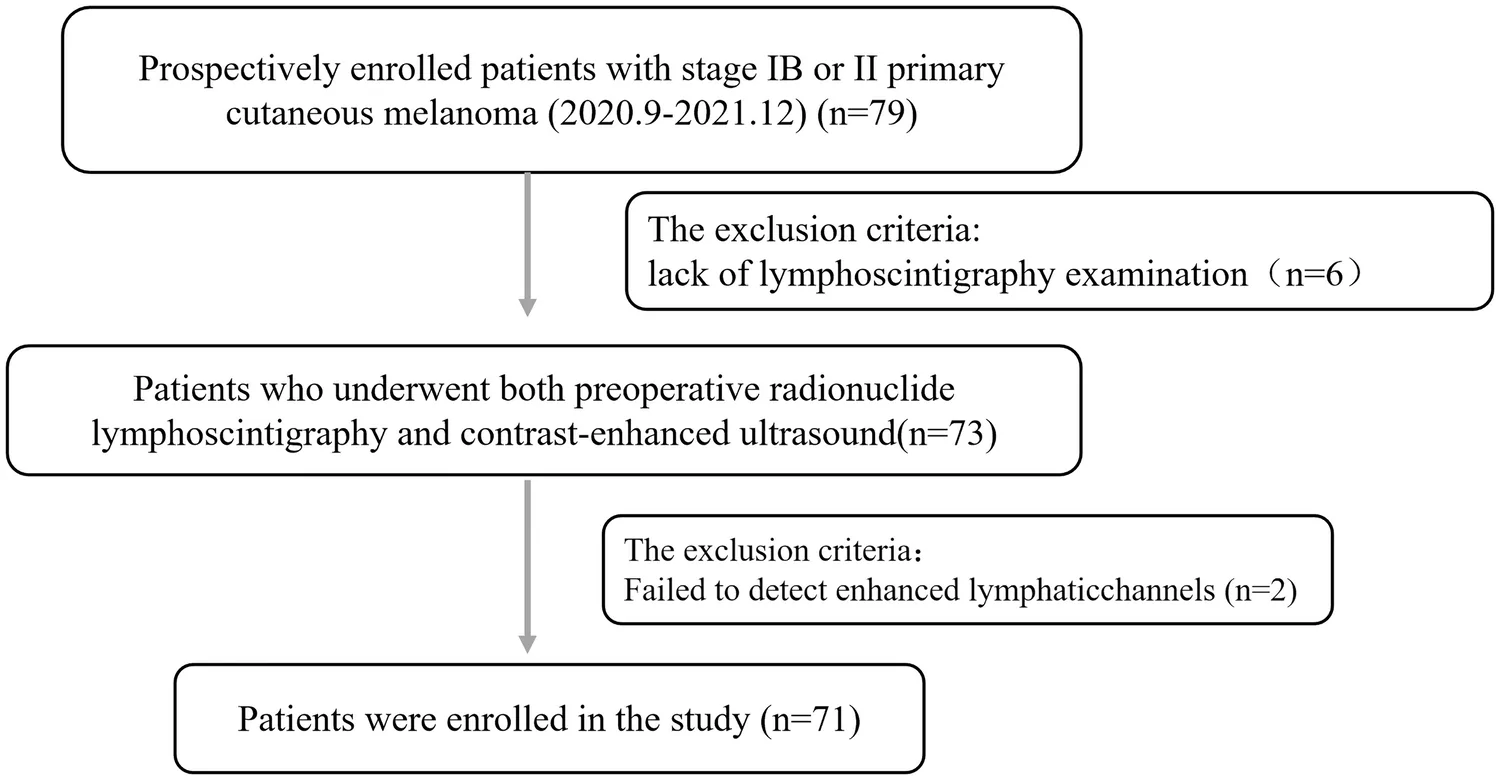
Flow diagram of patient selection. ALN, axillary lymph node; SLNB, sentinel lymph node biopsy; CEUS, contrast-enhanced ultrasound

From September 2020 to December 2021, a total of 79 patients with stage IB or II primary cutaneous melanoma were prospectively enrolled. Six patients were excluded because of the failure of radionuclide-based sentinel lymph node identification, resulting in 73 eligible patients. Among these, Sonazoid contrast-enhanced ultrasound (Sonazoid-CEUS) failed to identify sentinel lymph nodes in two patients, yielding a success rate of 97.3% (71/73). Among these two failed cases, one involved a shoulder melanoma that was pathologically confirmed to have no lymph node metastasis (0/1), while the other was a back melanoma that was confirmed to have metastatic involvement (1/1). Consequently, 71 patients were included in the final cohort (see the flowchart in Fig. 1). In 71 patients, the mean Breslow thickness of the melanoma lesions was 3.8 mm, with 43.7% (31/71) ulceration (Table 1).

**Table 1:** Patient and tumor characteristics

Characteristics	Patients	Percent (%)
Sex		
Male	33	46.5
Female	38	53.5
Age		
≤ 60	34	47.9
> 60	37	52.1
Breslow depth (mm)		
≤ 2.0	29	40.8
> 2.0	42	59.2
Clark level		
I	0	0
II	2	2.8
III	10	14.1
IV	47	66.2
V	12	16.9
Ulceration		
Present	31	43.7
Absent	40	56.3
HMB45		
Negative	35	49.3
Positive	36	50.7
Mitotic count		
< 1	28	39.4
≥ 1	43	60.6
Primary lesion location		
Distal limb	62	87.3
Proximal limb	1	1.4
Head and neck, trunk	8	11.3

### CEUS-identified SLNs

**Fig. 2:**
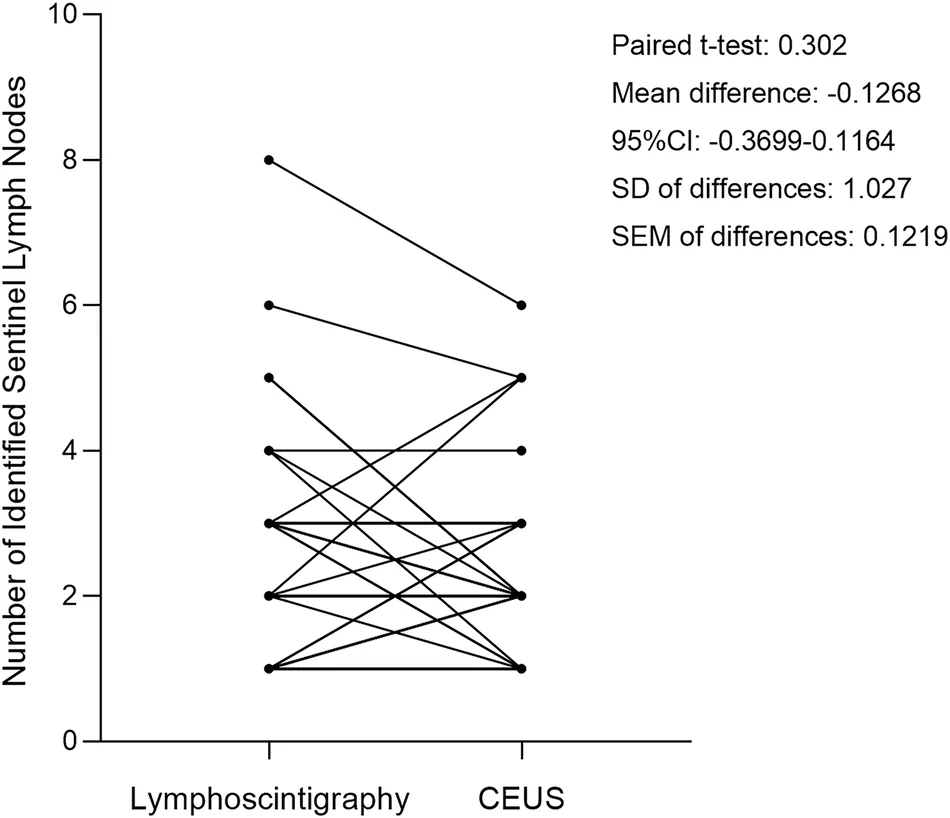
Comparison of the number of SLNs identified by preoperative CEUS and those identified by lymphoscintigraphy. SLN, sentinel lymph node; CEUS, contrast-enhanced ultrasound

**Fig. 3:**
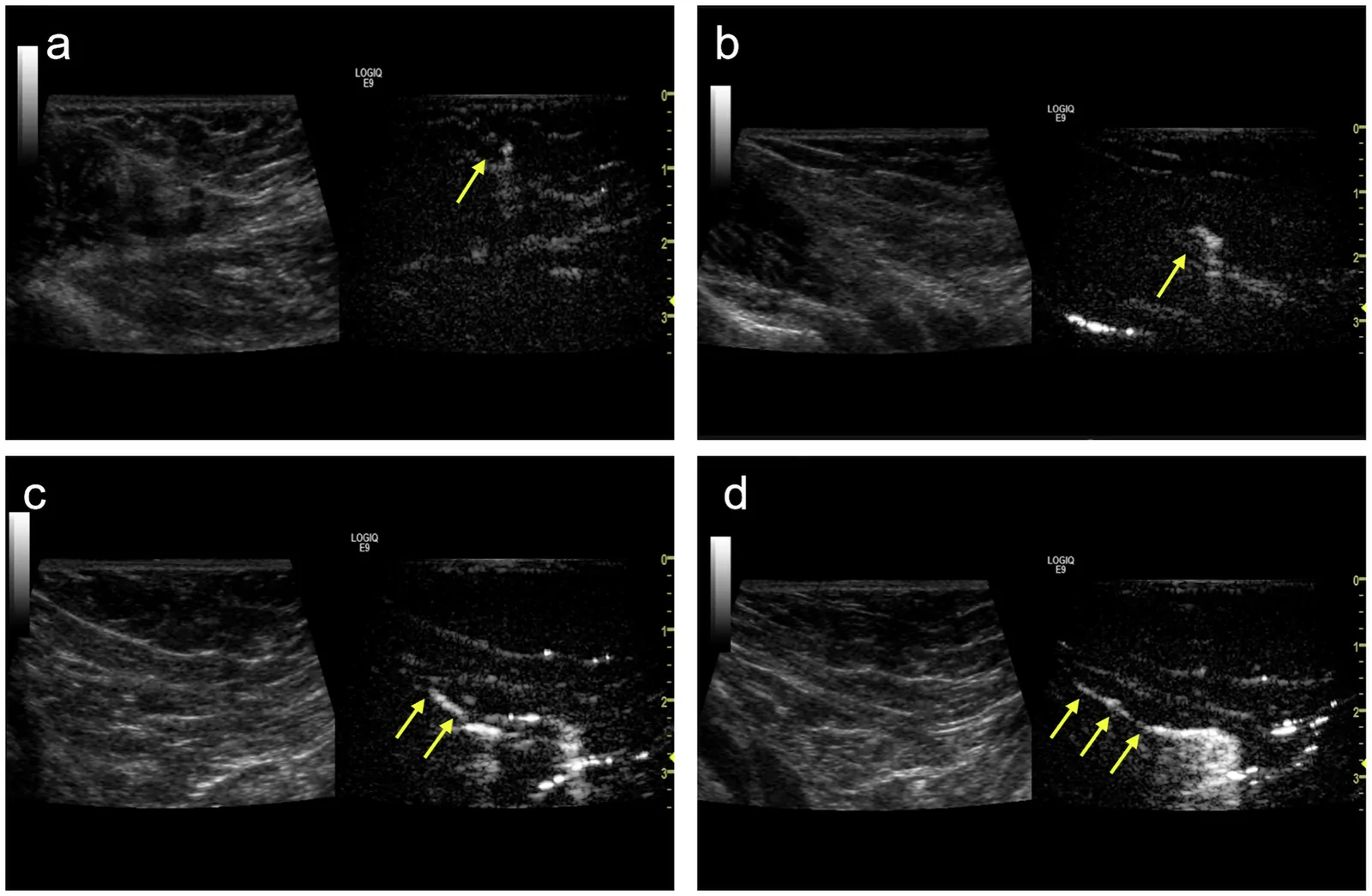
Real-time Sonazoid contrast-enhanced ultrasound (CEUS) for sentinel lymph node (SLN) mapping in a patient with cutaneous melanoma. **a**–**c** Serial CEUS images demonstrating the course of an enhancing sentinel lymphatic channel (SLC) following perilesional injection of the contrast agent. The SLC (arrows) originates from the injection site and passes through the intradermal tissue. **d** The SLC (arrow) is shown draining into and opacifying the sentinel lymph node (triangle), confirming its identity. The yellow arrows point to the enhanced lymphatic channels

CEUS identified 148 SLNs, with an average of 2.1 ± 1.1 SLNs per patient (range: 1–6), comparable to lymphoscintigraphy (Fig. 2). The distribution of the number of SLNs per patient was as follows: 21 patients had one SLN, 33 patients had two SLNs, 12 patients had three SLNs, one patient had four SLNs, three patients had five SLNs, and one patient had six SLNs. Representative real-time CEUS images demonstrating lymphatic channel enhancement and SLN identification (Fig. 3). With respect to lymphatic drainage patterns, 66 patients had SLNs in a single anatomical region, whereas five patients had SLNs distributed across two regions. The specific anatomical distribution of SLNs was as follows: groin region, 108 (73.0%); axillary region, 17 (11.5%); popliteal fossa, 18 (12.2%); and submandibular region, 5 (3.4%).

### SLNB of patients

Lymphoscintigraphy and SLNB yielded an equal number of detected SLNs in the patients. Lymphoscintigraphy revealed a total of 157 SLNs, with an average of 2.2 ± 1.3 SLNs per patient (range: 1–8). The distribution of the number of SLNs per patient was as follows: 23 patients had one SLN, 25 patients had two SLNs, 16 patients had three SLNs, three patients had four SLNs, two patients had five SLNs, one patient had six SLNs, and one patient had eight SLNs. With respect to lymphatic drainage patterns, 63 patients had SLNs in a single anatomical region, whereas eight patients had SLNs in two regions. The number of SLNs detected via CEUS (2.1 ± 1.1) did not significantly differ from that detected via lymphoscintigraphy (2.2 ± 1.3). As the paired differences in the SLN counts significantly deviated from a normal distribution (Shapiro–Wilk test, *p* < 0.001), the primary comparison was performed by using the non-parametric Wilcoxon signed-rank test, which demonstrated no significant difference between the two modalities (*p* = 0.305). A supplementary paired *t*-test yielded a consistent result (*p* = 0.302), thus confirming the robustness of the findings. These findings are summarized in Fig. 2. The specific anatomical distribution of SLNs was as follows: groin, 105 (66.9%); axilla, 20 (12.7%); popliteal fossa, 26 (16.6%); and submandibular region, 6 (3.8%).

### Diagnostic efficacy of CEUS

**Fig. 4:**
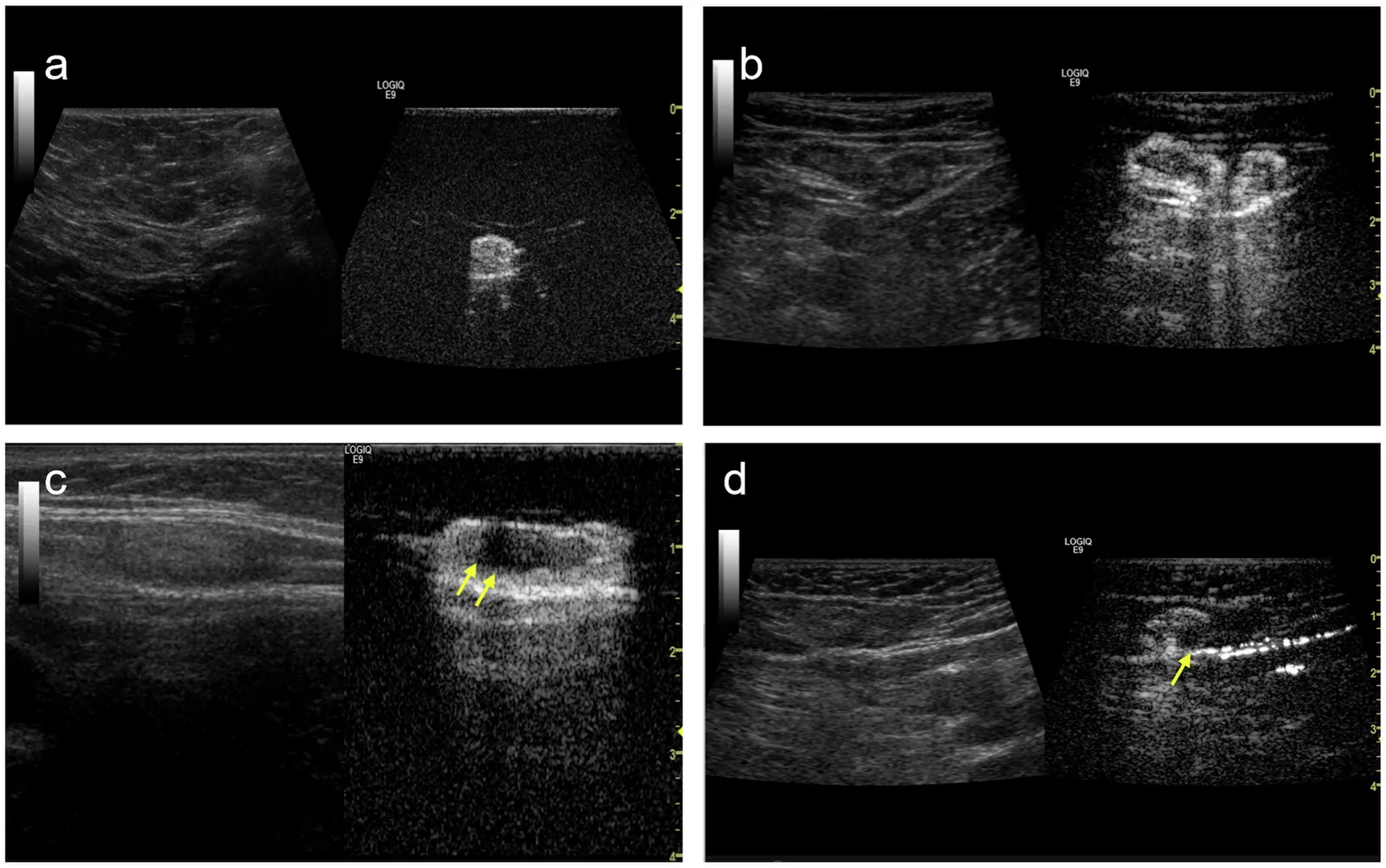
Sonazoid contrast-enhanced ultrasound patterns of sentinel lymph nodes in a patient with cutaneous melanoma. **a**, **b** Enhancement patterns of benign sentinel lymph nodes. **a** Homogeneous high enhancement is demonstrated. **b** Thin rim-like moderate enhancement is demonstrated. **c**, **d** Enhancement patterns of metastatic sentinel lymph nodes. The images show characteristic contrast perfusion defects in the local cortex (yellow arrows), suggesting tumor infiltration

Typical contrast-enhanced ultrasound features of benign and metastatic SLNs are shown in Fig. 4. Among the 71 patients, 28 were confirmed to have lymph node metastases upon pathology. Of the 157 harvested lymph nodes, 48 were metastatic. The distribution of metastatic nodes among patients was as follows: 14 patients had one metastatic node, 9 patients had two, 4 patients had three, and 1 patient had four.

**Fig. 5:**
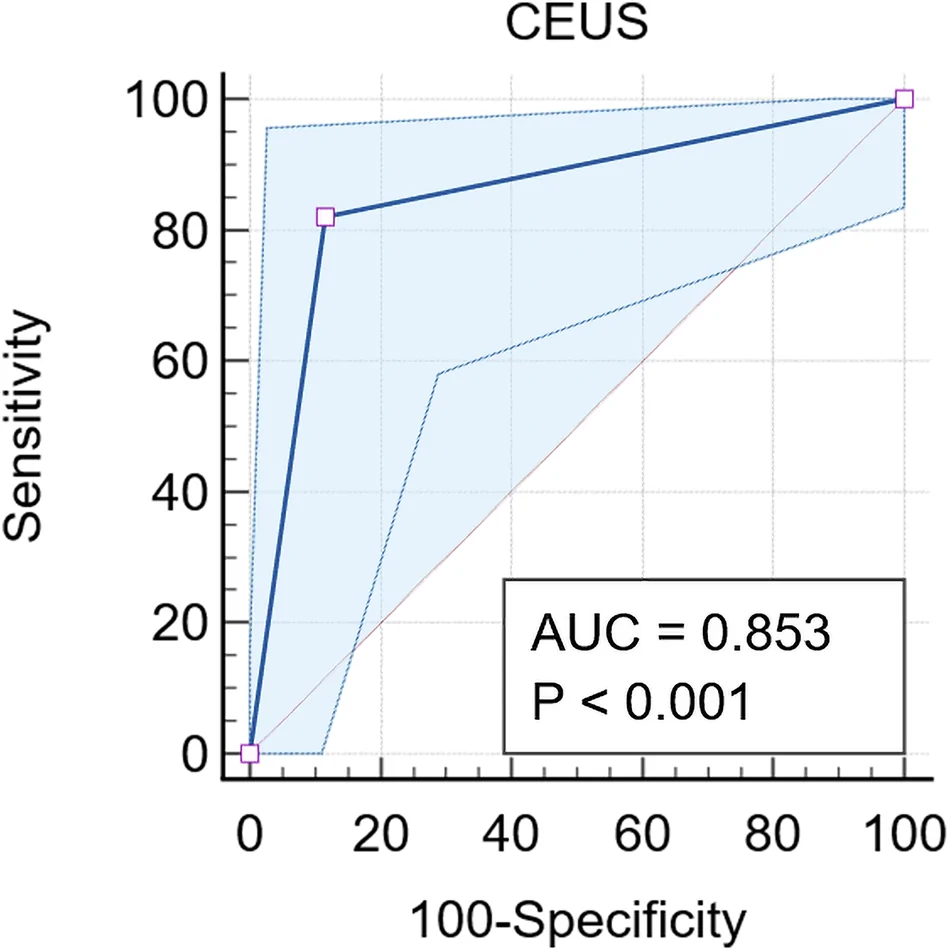
Diagnostic accuracy of contrast-enhanced ultrasound

Contrast-enhanced ultrasound diagnosed lymph node metastases in 28 of the 71 patients. Among the 148 identified lymph nodes, 38 were diagnosed as metastatic. The distribution was as follows: 19 patients were diagnosed with one metastatic node, 8 patients with two, and 1 patient with three. The diagnostic performance yielded a sensitivity of 82.1%, a specificity of 88.4%, and an accuracy of 85.9% (Fig. 5).

### Influence factor analysis of Sonazoid-CEUS and SLN metastasis

The number of SLNs detected via Sonazoid-CEUS was observed to be higher in patients with primary lesions located in the distal limb [2.0 (1.8–3.0)] than in the proximal limb, as well as the head, neck and trunk [1.0 (1.0–2.0), *p* < 0.001]. The SLN metastatic rates were higher in patients with ulcerations (*p* = 0.005), mitotic counts (*p* = 0.045) and HMB45 expression (*p* < 0.01), as detailed in Table 2.

**Table 2:** Relationships between the clinical pathological characteristics of patients with cutaneous melanoma with Sonazoid-CEUS and with SLNB

Characteristics	Patients	Sonazoid-CEUS		SLNB		SLN pathology	
		SLNs, median (IQR)	*p*-value	SLNs, median (IQR)	*p*-value	Metastatic SLNs (%)	*p*-value
Sex					0.820		0.327
Male	33	2.0 (1.0–2.0)	0.090	2.0 (1.0–3.0)		11 (33.3)	
Female	38	2.0 (1.0–3.0)		2.0 (1.0–3.0)		17 (44.7)	
Age			0.118		0.885		0.242
≤ 60	34	2.0 (1.0–2.0)		2.0 (1.0–3.0)		11 (32.4)	
> 60	37	2.0 (1.5–3.0)		2.0 (1.0–3.0)		17 (45.9)	
Breslow depth(mm)			0.773		0.817		0.378
≤ 2.0	29	2.0 (1.0–2.5)		2.0 (1.0–3.0)		10 (34.5)	
> 2.0	42	2.0 (1.0–2.3)		2.0 (1.0–3.0)		18 (42.9)	
Clark level			0.928		0.829		0.753
I–III	12	2.0 (1.3–2.0)		2.0 (1.0–3.8)		4 (33.3)	
IV–V	59	2.0 (1.0–3.0)		2.0 (1.0–3.0)		24 (40.7)	
Ulceration			0.911		0.681		0.005
Present	31	2.0 (1.0–3.0)		2.0 (1.0–3.0)		18 (58.1)	
Absent	40	2.0 (1.0–2.0)		2.0 (1.0–3.0)		10 (25.0)	
HMB45			0.090		0.615		< 0.001
Negative	35	2.0 (2.0–3.0)		2.0 (1.0–3.0)		3 (8.6)	
Positive	36	2.0 (1.0–2.0)		2.0 (1.0–3.0)		25 (69.4)	
Mitotic count			0.930		0.722		0.045
< 1	28	2.0 (1.0–2.0)		2.0 (1.0–3.0)		7 (25.0)	
≥ 1	43	2.0 (1.0–3.0)		2.0 (1.0–3.0)		21 (48.8)	
Primary lesion location			0.024		0.766		0.732
Distal limb	62	2.0 (1.8–3.0)		2.0 (1.0–3.0)		38 (62.3)	
Proximal limb, head and neck, trunk	9	1.0 (1.0–2.0)		2.0 (1.0–3.0)		4 (44.4)	

*IQR* interquartile range, *SLNB* sentinel lymph node biopsy

### Safety

No serious side effects associated with the contrast agent Sonazoid were documented following its percutaneous injection.

## Discussion

The accurate identification of sentinel lymph nodes (SLNs) is essential for accurate staging and optimal management of patients with cutaneous melanoma [4, 28]. In this prospective study, we demonstrated that contrast-enhanced ultrasound using Sonazoid is a safe and effective modality for SLN identification and preoperative nodal assessment in patients with stage IB–II melanoma. Sonazoid-CEUS achieved a high SLN identification success rate and encouraging diagnostic accuracy, with no contrast-related adverse events being observed, thus supporting its feasibility and safety within the clinical workflow of melanoma SLN mapping. These findings suggest that Sonazoid-CEUS may serve as a complementary imaging modality within established SLNB workflows.

The SLN identification success rate of 97.3% observed in this study is comparable to the rates previously reported for other ultrasound contrast agents (such as SonoVue) in melanoma [18, 19]. Although prior studies [19, 29] have confirmed the feasibility and favorable diagnostic accuracy of SonoVue-based CEUS for SLN mapping, its clinical application is constrained by its mechanism of action as a purely intravascular blood-pool agent with short intravascular persistence and no specific tissue retention [30]. Consequently, imaging must be rapidly performed after injection, which may limit the available time for meticulous tracking of lymphatic channels, particularly in cases with slow or ambiguous lymphatic drainage. In contrast, Sonazoid microbubbles are phagocytosed by macrophages within the nodal reticuloendothelial system [22, 31], thus resulting in prolonged retention within lymph node sinuses and sustained enhancement [32]. This unique pharmacokinetic behavior extends the practical imaging window and provides more stable visualization of SLNs, thereby potentially improving the consistency of SLN identification and confidence in interpreting nodal perfusion patterns. Although both agents offer non-radioactive CEUS approaches, the prolonged nodal retention associated with Sonazoid may confer practical advantages for preoperative SLN localization, particularly when delayed imaging is required, although direct head-to-head comparative studies remain limited.

It is important to acknowledge that the generalizability of our findings is influenced by the anatomical distribution of the study cohort. The majority of primary melanomas were located on the distal limbs, thus resulting in most SLNs being identified within the inguinal or popliteal basins. Although this relatively predictable lymphatic drainage pattern provided a favorable model for initial technical validation of Sonazoid-CEUS, it inherently limits extrapolation of the high detection rate and predominantly single-basin drainage pattern to melanomas arising in the head, neck, or trunk. Lymphatic drainage from these regions is well known to be more complex and variable, with a higher frequency of multiple or interval nodal basins being reported [33–35]. Therefore, the performance metrics reported in this study should be interpreted as primarily applicable to distal limb melanomas, and further evaluation in anatomically complex regions is warranted.

From a clinical workflow perspective, the number of SLNs identified per patient and the overall drainage patterns observed with CEUS were highly concordant with those obtained using lymphoscintigraphy [36]. This concordance supports the reliability of CEUS in delineating lymphatic anatomy and predicting SLN location, thus offering surgeons a valuable preoperative “roadmap” that may facilitate incision planning, reduce operative time, and minimize tissue dissection. However, it should be noted that although this study focused on an ultrasound-based technique, blue dye remains a widely used visual adjunct in many surgical practices worldwide [13–15] and continues to play an important role within established SLNB workflows.

With respect to metastatic assessment, CEUS demonstrated robust diagnostic performance with high specificity and overall accuracy, which is consistent with previous studies [19, 37]. However, due to the fact that CEUS primarily reflects lymphatic tracer dynamics rather than microscopic tumor burden, small-volume metastases may remain morphologically and functionally inconspicuous [38]. Prior reports [29, 37] have demonstrated that false-negative CEUS findings are frequently associated with micrometastases (< 2 mm), which may still exhibit homogeneous enhancement due to preserved nodal architecture. In addition, subtle lymphatic filling and deeply located or small lymph nodes can further reduce lesion conspicuity on ultrasound, thus representing recognized technical limitations of lymphatic CEUS [39]. These biological and imaging factors likely explain the limited discordance between CEUS and histopathology observed in a minority of cases.

Several limitations of this study should be acknowledged. First, this was a single-center study with a relatively limited sample size and a predominance of distal limb melanomas, which introduces selection bias and restricts generalizability to other anatomical regions. Second, CEUS was not evaluated as a fully independent diagnostic modality. Moreover, its performance was assessed within a conventional SLNB pathway that incorporated lymphoscintigraphy; thus, its diagnostic accuracy is partially dependent on the existing workflow. Third, early dynamic lymphoscintigraphy was not performed, thus limiting direct verification of lymphatic drainage sequence and nodal echelon differentiation [40]. Finally, post-surgical node-by-node confirmation between CEUS-identified SLNs and lymphoscintigraphy-identified SLNs was not performed. Future multicentre studies with larger and more anatomically diverse cohorts incorporating dynamic reference imaging and direct nodal correlation are needed to further define the independent and complementary roles of Sonazoid-CEUS.

In conclusion, this study demonstrates that Sonazoid-enhanced CEUS is a safe and effective technique for the identification and preoperative evaluation of sentinel lymph nodes in patients with cutaneous melanoma. The unique phagocytic uptake and prolonged nodal retention of Sonazoid contribute to a high SLN detection rate and a practical imaging window. At present, Sonazoid-CEUS should be regarded as a complementary imaging modality within established SLNB workflows rather than a standalone replacement for lymphoscintigraphy, pending validation in larger comparative studies.

## Abbreviations

CEUS: Contrast-enhanced ultrasound
H&E: Hematoxylin and eosin
IQR: Interquartile range
SLC: Sentinel lymphatic channel
SLN: Sentinel lymph node
SLNB: Sentinel lymph node biopsy
Sonazoid-CEUS: Contrast-enhanced ultrasound using Sonazoid
SPECT/CT: Single-photon emission computed tomography/computed tomography
